# Enhancing neutralization of *Plasmodium falciparum* using a novel monoclonal antibody against the rhoptry-associated membrane antigen

**DOI:** 10.1038/s41598-022-06921-1

**Published:** 2022-02-23

**Authors:** Anne S. Knudsen, Melanie R. Walker, Judit P. Agullet, Kasper H. Björnsson, Maria R. Bassi, Lea Barfod

**Affiliations:** Department of Immunology and Microbiology, Centre for Medical Parasitology, Faculty of Health and Medical Sciences, University of Copenhagen, Copenhagen, Denmark

**Keywords:** Immunology, Infectious diseases, Malaria

## Abstract

The pathogenesis of malaria is associated with blood-stage infection and there is strong evidence that antibodies specific to parasite blood-stage antigens can control parasitemia. This provides a strong rational for applying blood-stage antigen components in a multivalent vaccine, as the induced antibodies in combination can enhance protection. The *Plasmodium falciparum* rhoptry-associated membrane antigen (PfRAMA) is a promising vaccine target, due to its fundamental role in merozoite invasion and low level of polymorphism. Polyclonal antibodies against PfRAMA are able to inhibit *P. falciparum* growth and interact synergistically when combined with antibodies against *P. falciparum* reticulocyte-binding protein 5 (PfRh5) or cysteine-rich protective antigen (PfCyRPA). In this study, we identified a novel PfRAMA-specific mAb with neutralizing activity, which in combination with PfRh5- or PfCyRPA-specific mAbs potentiated the neutralizing effect. By applying phage display technology, we mapped the protective epitope to be in the C-terminal region of PfRAMA. Our results confirmed previous finding of synergy between PfRAMA-, PfRh5- and PfCyRPA-specific antibodies, thereby paving the way of testing these antigens (or fragments of these antigens) in combination to improve the efficacy of blood-stage malaria vaccines. The results emphasize the importance of directing antibody responses towards protective epitopes, as the majority of anti-PfRAMA mAbs were unable to inhibit merozoite invasion of erythrocytes.

## Introduction

Malaria caused by *Plasmodium falciparum* parasites remains a significant global health challenge, causing more than 200 million cases and 409,000 deaths in 2019^1^. The clinical manifestations of disease result from repeated cycles of merozoite invasion, replication within and lytic egress from erythrocytes. The invasion process involves a highly regulated discharge of four secretory organelles, rhoptries, micronemes, exonemes and dense granules, which are positioned in the apical end of the merozoite^2^. Some organelle components are discharged on the merozoite surface prior to invasion, while others are discharged once the merozoite recognizes and forms a tight junction between its apical pole and the erythrocyte membrane^3^. Apical organelles, like the rhoptries, are formed de novo during each asexual life cycle, suggesting a transient role of their molecular components during merozoite invasion^4,5^.

The largest of the apical organelles, the rhoptries, are characterized by a wide bulb region that narrows to a neck towards the apical end of the merozoite. Rhoptries contain both protein and lipid components. To date, multiple rhoptry proteins have been identified, for instance the high-molecular weight (HMW) proteins that form a complex involving PfRhopH1/Clag, PfRhopH2 and PfRhopH3. These proteins are important for erythrocyte invasion and nutrient uptake during *P. falciparum* blood-stage development^6,7^. The identification of the low molecular weight (LMW) PfRAP1/PfRAP2 protein complex has been implicated in formation of the parasitophorous vacuole membrane^8^ and essential rhoptry neck proteins like PfRON2, PfRON4 and PfRON5 form a tight junction complex with the micronemal protein, PfAMA1^9^. The complex proteins, in particular PfAMA1, has previously been highlighted as a key blood-stage malaria vaccine target^10^.

Another important rhoptry protein is PfRAMA, which is expressed relatively early in the blood-stage cycle, even prior to rhoptry formation^11,12^. PfRAMA is attached to the inner face of the rhoptry bulb via its glycosyl phosphatidylinositol (GPI) membrane anchor^11^. It is expressed as a 170 kDa PfRAMA precursor, which later undergoes proteolytic cleavage in the rhoptries, which removes the N-terminal segment to produce a mature C-terminal form of 60 kDa^11^. By generating a PfRAMA conditional knockdown parasite line, it was shown that protein disruption does not affect blood-stage development, but causes severe defect of erythrocyte invasion^13^. Studies have indicated that PfRAMA interacts with other rhoptry proteins, for instance PfRhop3, PfRAP1 and PfRAP2^11,14^. However, recent data contradict these findings, as PfRAMA was shown only to interact with the rhoptry neck proteins PfRON1, PfRON2 and PfRON3^13,15^, indicating that loss of PfRAMA leads to mislocalization or loss of the rhoptry neck proteins, and as a result generates ‘neckless’ rhoptries^13^.

Although the rhoptry protein PfRAMA is only accessible to antibodies transiently, numerous sero-epidemiological studies have demonstrated the development of IgM and IgG responses to the antigen^16–19^. Interestingly, antibodies towards C-terminal PfRAMA have been associated with protection against malaria^16,19^. Furthermore, this region contains an erythrocyte binding domain^11^, suggesting that directing an antibody response against C-terminal PfRAMA might confer higher levels of protection compared to the full-length protein. Antibodies specific to PfRAMA are able to inhibit in vitro growth of *P. falciparum* parasites, and a potentiating effect is observed when they are combined with antibodies specific to PfRh5 or PfCyRPA^17^. Another attractive feature of PfRAMA is its low antigenic diversity^20^, which in combination with its important role in merozoite invasion and rhoptry biogenesis, makes it a strong blood-stage vaccine candidate^13^.

In this study, we examined parasite neutralization using a panel of PfRAMA-specific mAbs to identify protective epitopes on the protein. The vast majority of anti-PfRAMA mAbs were unable to inhibit *P. falciparum* growth, however one mAb showed neutralizing activity at high concentrations and was able to synergize when combined with mAbs against the merozoite invasion proteins PfRh5 and PfCyRPA. The growth inhibitory anti-PfRAMA mAb recognized only certain segments of endogenously processed PfRAMA, suggesting that a protective epitope is present in the C-terminus of the protein. These findings emphasize the importance of directing the antibody response towards protective epitopes, as the majority of anti-PfRAMA mAbs did not confer any protection against *P. falciparum* invasion of erythrocytes.

## Results

### Expression and purification of recombinant PfRAMA

**Figure 1:**
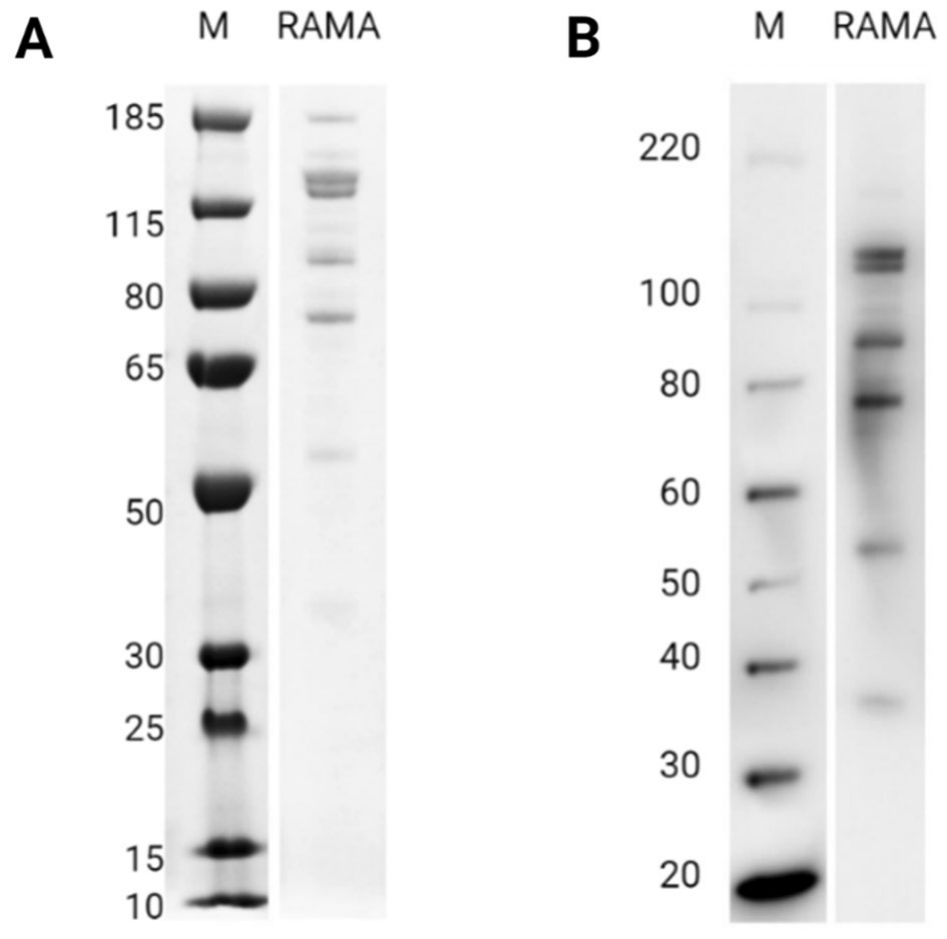
Assessing the quality of PfRAMA. **(A)** 1 µg of recombinant PfRAMA was resolved under non-reducing conditions by SDS-PAGE followed by Instant Blue staining. The expected size is 124 kDa including the tag-region (25 kDa). **(B)** Western blotting analysis of 100 ng of recombinant PfRAMA under non-reducing conditions, resolved by SDS-PAGE, blotted and detected using anti-his(C-term)-HRP conjugated antibody. Unprocessed images can be found in supplementary information Fig. S1.

PfRAMA-bio-his was expressed in Expi293F cells as a secreted protein. The supernatant was harvested three days post-transfection to avoid formation of aggregates and the protein was subsequently purified by IMAC chromatography. To assess the quality of the purified protein, PfRAMA was resolved by SDS-PAGE under non-reducing conditions and detected by InstantBlue staining (Fig. 1A and Figure S1A). A double band was present at the expected size of 124 kDa, as well as four lower molecular weight bands at 90-, 75-, 55- and 35 kDa respectively^21^. The smaller bands were also detected by western blotting, confirming that they are by-products of a proteolytic cleavage of the recombinant protein (Fig. 1B and Fig. S1B). No band was present at 25 kDa, which otherwise could have suggested cleavage of the C-terminal tag-region. Thus, the proteolytic cleavage seems to occur within the PfRAMA sequence. As the same bands were detected by InstantBlue staining and western blotting, the N-terminal part of PfRAMA seems to degrade rapidly upon processing.

### Anti-RAMA mAbs recognize endogenously expressed PfRAMA

Hybridoma technology was applied to produce PfRAMA-specific mAbs. Mice were immunized with recombinant PfRAMA-bio-his emulsified in the adjuvant AddaVax. This was followed by performing a PfRAMA-specific serum ELISA to identify the best immune responders, after which splenocytes of an immunized mouse were fused with myeloma cells to generate hybridoma cell lines. By screening cell supernatants by ELISA, a panel of 13 anti-PfRAMA mAbs was generated.

**Figure 2:**
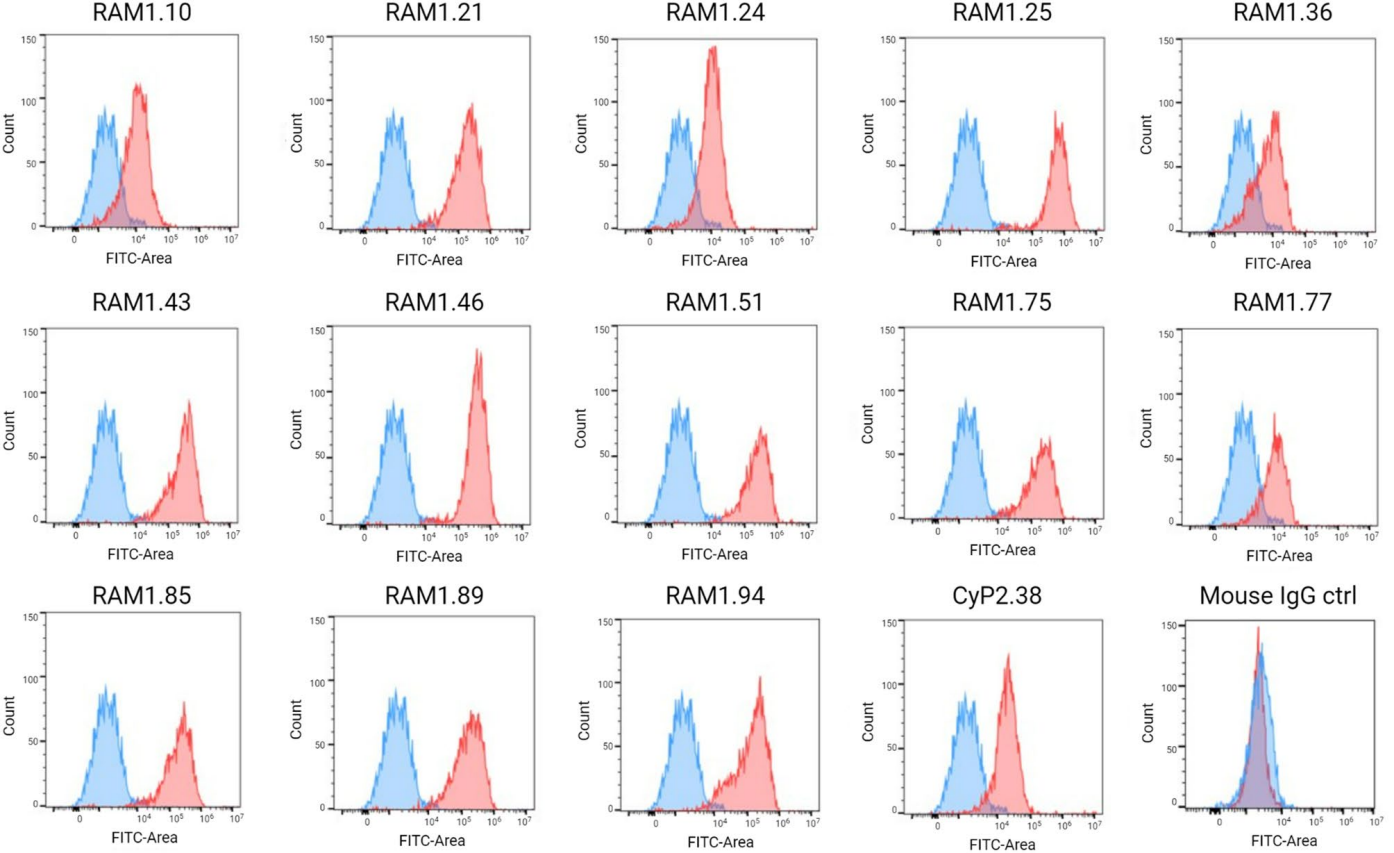
Flow cytometry analysis of anti-PfRAMA mAbs binding to *P. falciparum* merozoites. The histograms (red curves) show each of the anti-PfRAMA mAbs binding to 3D7 merozoites after detecting the mAbs with FITC conjugated anti-mouse IgG. The blue curve represent the negative control generated by adding the detection antibody only. The anti-PfCyRPA mAb (CyP2.38) was included as a positive control and non-malarial specific mouse IgG was included as a negative control. The histograms were generated by gating on size/granularity, single cells and positive nuclei DAPI signal.

As recombinant PfRAMA seemed to generate significantly more processed fragments than the endogenous protein^11,21^, we began our assessment by determining whether the anti-PfRAMA mAbs were capable of detecting the protein in *P. falciparum* merozoites using flow cytometry. Due to the intracellular location of the protein, merozoites were fixed and permeabilized prior to adding the anti-PfRAMA mAbs. The flow cytometry analysis indicated that all anti-PfRAMA mAbs bind the endogenously expressed protein (Fig. 2). The fluorescence intensity varied between the anti-PfRAMA mAbs, which could be explained by different affinities or epitope specificities. As PfRAMA comprises three distinct repeat regions of charged residues^11^, it is possible that the mAbs recognize multiple segments, or that some epitopes are disrupted more than others, due to the fixation and permeabilization treatments of the merozoites.

### Neutralizing activity of anti-PfRAMA mAbs

**Figure 3:**
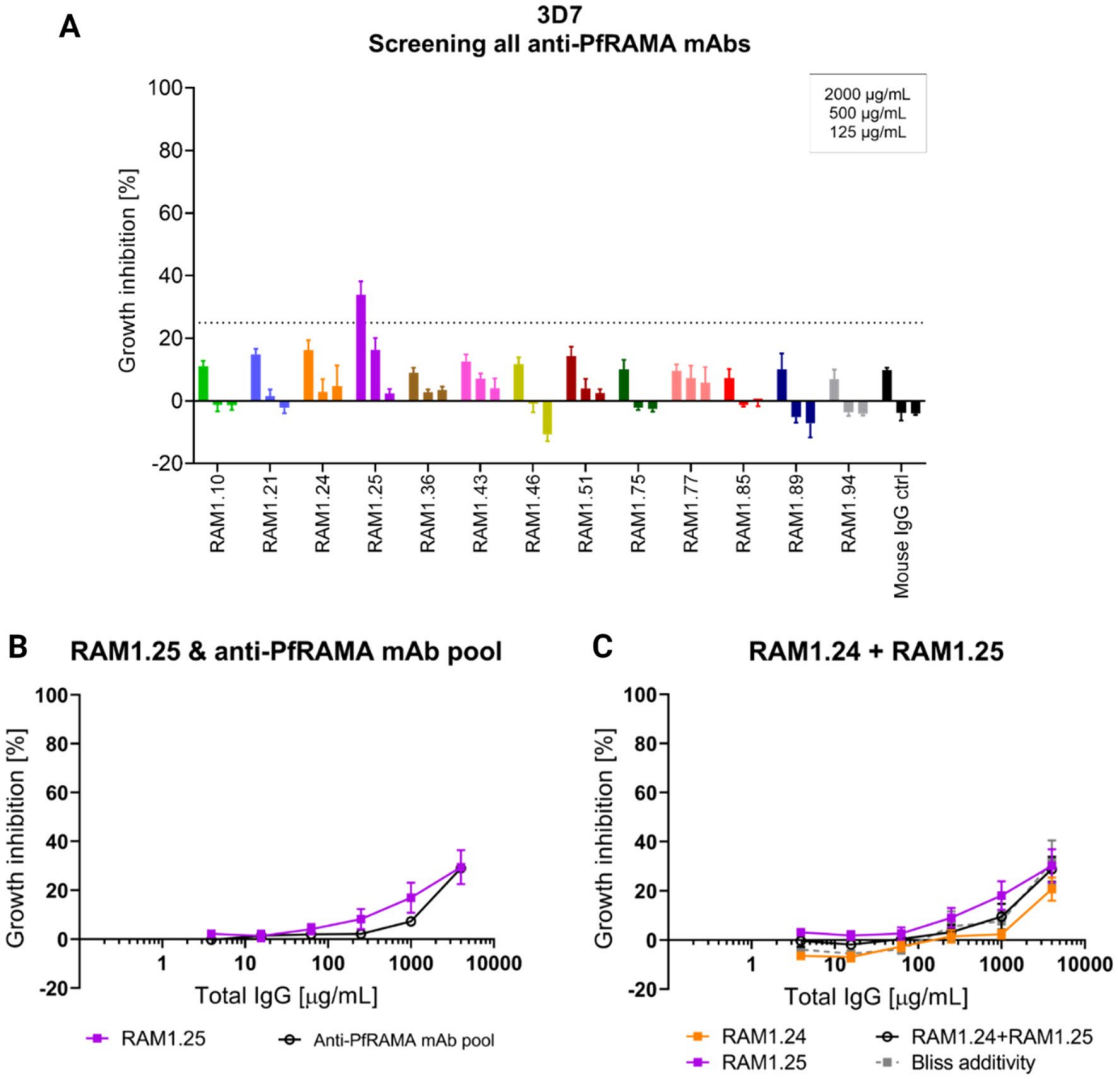
Growth inhibition activity of PfRAMA mAbs. **(A)** GIA assay using a panel of PfRAMA-specific mAbs screened at 2000, 500 and 125 µg/mL against *P. falciparum* 3D7 strain. A black dotted line shows a cut-off between mAbs inducing high GIA and mAbs inducing negligible GIA. **(B)** GIA assay using dilution series of RAM1.25 and a pool of 13 anti-PfRAMA mAbs, starting at 4000 µg/mL, against 3D7 parasites. **(C)** GIA assay using a dilution series of individual mAbs (RAM1.24 and RAM1.25) as well as in combination against 3D7 parasites, all starting at 4000 µg/mL. The predicted additive effect was calculated using the formula of Bliss additivity shown in the grey dotted line. The observed effect of combining the two anti-PfRAMA mAbs is shown in the black solid line. Error bars show SEM for six replicates over two experiments.

The anti-PfRAMA mAbs were screened for their ability to block merozoite invasion into erythrocytes using the in vitro GIA assay against *P. falciparum* 3D7 parasites. The mAbs were tested at three concentrations, 2000, 500 and 125 µg/mL, with a cut-off at 25% GIA (Fig. 3A). Only one mAb, RAM1.25, showed detectable levels of neutralization. Next, a GIA assay against 3D7 parasites was setup to compare the effect of RAM1.25 and of a pool of all 13 anti-PfRAMA mAbs starting at 4000 µg/mL. The pool of anti-PfRAMA mAbs was made by combining the mAbs in equimolar ratios in the GIA assay to explore whether targeting several epitopes on PfRAMA simultaneously could potentiate the neutralizing effect, as this have been observed with mAbs against other blood-stage antigens^22–24^. However, the result did not show any improvement in GIA by this approach, as the neutralizing effect of the pooled mAbs was similar to RAM1.25 on its own (Fig. 3B). In a last attempt to potentiate the GIA effect by targeting PfRAMA, the two most promising mAbs (RAM1.24 and RAM1.25) were mixed at an equimolar ratio against 3D7 parasites (Fig. 3C). The predicted additive GIA effect was calculated by the definition of Bliss additivity. This was followed by applying a 2-way ANOVA test to assess whether the observed and predicted GIA values of the mAb combination resulted in synergy, additivity or antagonism^25^. Again, no improvement in GIA was observed when combining anti-PfRAMA mAbs.

### An anti-PfRAMA mAb synergize when combined with mAbs against PfRh5 and PfCyRPA

**Figure 4:**
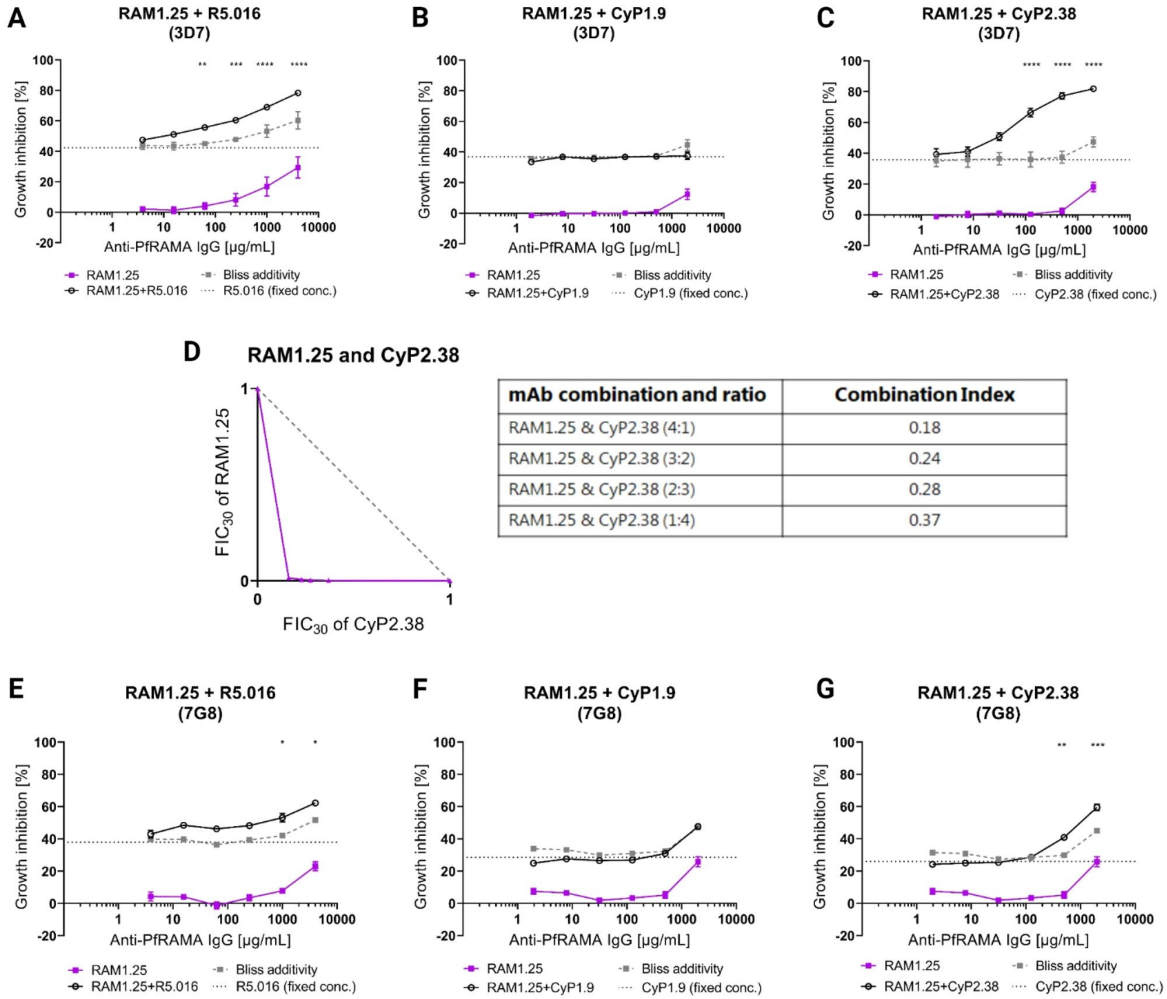
Growth inhibition activity of anti-PfRAMA mAb in combination with anti-PfRh5 or anti-PfCyRPA mAbs. **(A)** GIA assay using a dilution series of RAM1.25, starting at 4000 µg/mL, mixed with a fixed concentration of anti-PfRh5 mAb (R5.016, 2 µg/mL) against *P. falciparum* 3D7. **(B)** GIA assay investigating the effect of combining RAM1.25, starting at 2000 µg/mL, with a fixed concentration of the anti-PfCyRPA mAb (CyP1.9, 10 µg/mL) against 3D7 parasites. **(C)** Similar GIA assay, exploring the combination of RAM1.25 and a fixed concentration of the anti-PfCyRPA mAb (CyP2.38, 40 µg/mL). **(D)** Isobologram analysis of the RAM1.25 and CyP2.38 mAb combination using fixed ratio mixtures of (5:0, 4:1, 3:2, 2:3, 1:4 and 0:5) against 3D7 parasites. The dashed grey line indicates the predicted results following the definition of Loewe additivity. The purple line links the observed values of each explored mAb ratio that induces 30% GIA. Points below the line of additivity indicate synergistic interactions between the mAbs, while points above indicate antagonistic interactions. Each point shows the mean of two independent experiments performed in triplicates. The combination index (CI) of the RAM1.25 and CyP2.38 combination, was calculated as the sum of the two FIC_30_ values. CI < 1, CI = 1 or CI > 1 respectively indicate synergy, additivity or antagonism. **(E)** GIA assay against 7G8 parasites using the mAb combination of RAM1.25 and a fixed concentration of R5.016 (5 µg/mL). GIA assay against 7G8 parasites, respectively investigating the effect of combining RAM1.25 with a fixed concentration of CyP1.9 (8 µg/mL) **(F)** or of combining RAM1.25 with a fixed concentration of CyP2.38 (150 µg/mL) **(G)**. The formula of Bliss additivity was applied to calculate the predicted additive effect of a specific mAb combination, shown in the grey dotted line. The observed effect of combining two mAbs is shown in the solid black line. Bars show the SEM for six replicates over two experiments. The asterisks indicate where the experimental and the predicted GIA values of the mAb combination significantly show synergy, calculated using a 2-way ANOVA with Bonferroni’s multiple comparison test (*p < 0.05, **p < 0.01, ***p < 0.001, ****p < 0.0001).

As polyclonal antibodies against PfRAMA have been shown to inhibit *P. falciparum* growth^17^, we were surprised that the vast majority of our anti-PfRAMA mAbs showed no GIA, despite that the flow cytometry analysis had indicated detection of endogenous PfRAMA. Thus, we extended the functional analysis by investigating the effect of combining RAM1.25 with mAbs targeting the essential merozoite invasion proteins PfRh5 or PfCyRPA^22,23^ These mAbs were accessible to use, as they either had been generated in our own research group or by collaborators. The formula of Bliss additivity was applied to assess the predicted additive GIA effect of a given mAb combination. The anti-PfRAMA mAb (RAM1.25) was mixed with a fixed concentration of the very potent anti-PfRh5 mAb (R5.016)^22^, resulting in roughly 40% GIA on its own. An enhanced neutralization of 3D7 parasites was observed, indicating a clear synergistic interaction between the two mAbs (Fig. 4A). Similarly, a dilution series of RAM1.25 was mixed with a fixed concentrations of two different neutralizing anti-PfCyRPA mAbs, CyP1.9 or CyP2.38^23^, and screened in the GIA assay against 3D7 parasites (Fig. 4B,C). Remarkably, a potentiated neutralizing effect was observed only between RAM1.25 and CyP2.38, which might be explained by the anti-PfCyRPA mAbs unique epitope and high association rate^23^. In our GIA assay, we primarily employed the LDH assay as a readout. However, the inhibitory activity of some of our mAbs were assessed by both LDH and light microscopy (Figure S2). This comparative assessment indicated only minor differences between the two read-outs, which also has been demonstrated previously^26,27^.

We further assessed the effect of combining RAM1.25 and CyP2.38 by an isobologram analysis, which applies the definition of Loewe additivity to assess the effect between drugs^25,28^. The antibody interactions were investigated over a range of concentrations by a fixed-ratio method^29^. As the RAM1.25 mAb only exhibits low inhibitory activity at 2000 µg/mL, the EC_30_ values were interpolated from the dose–response curves as opposed to EC_50_ values. These values were converted to FIC_30_ values, and for each fixed concentration ratio, FIC_30_ values were applied to construct an isobologram. For the RAM1.25:CyP2.38 mAb combination, the isobologram showed that the concentration of antibody required to inhibit 30% of parasites, is lower than what would be predicted by Loewe additivity (Fig. 4D). The observed effect of the mAb combination deviated strongly below the line of additivity, indicating synergistic interactions, which was also demonstrated by the combination indexes. Thus, the combination of RAM1.25 and CyP2.38 was clearly synergistic regardless of which method was used to define synergy.

After having observed these clear synergistic interactions against 3D7 parasites, we wanted to widen our functional analysis by exploring the effect against another *P. falciparum* strain. Thus, GIA assays were setup against 7G8 parasites, which contain no polymorphisms in PfCyRPA, one polymorphism in PfRh5 (C203Y) and the following polymorphisms in PfRAMA (T196A, V315E, E320Q, D323Y, Q325E, M326F, E389Q, K734E, D737N and an insertion 328–333 NEEFKN). The GIA assays were performed in a similar fashion as against 3D7 parasites, using a fixed concentration of the more potent mAb and a dilution series of RAM1.25. Again, synergy was observed at high concentrations between RAM1.25 and the anti-PfRh5 mAb (R5.016) (Fig. 4E). The pattern also remained the same using the combinations of RAM1.25 and anti-PfCyRPA mAbs, as the RAM1.25 and CyP1.9 interaction was additive (Fig. 4F) and the RAM1.25 and CyP2.38 was synergistic at high concentrations (Fig. 4G) using the definition of Bliss additivity.

### Characterizing the epitopes of anti-PfRAMA mAbs

**Figure 5:**
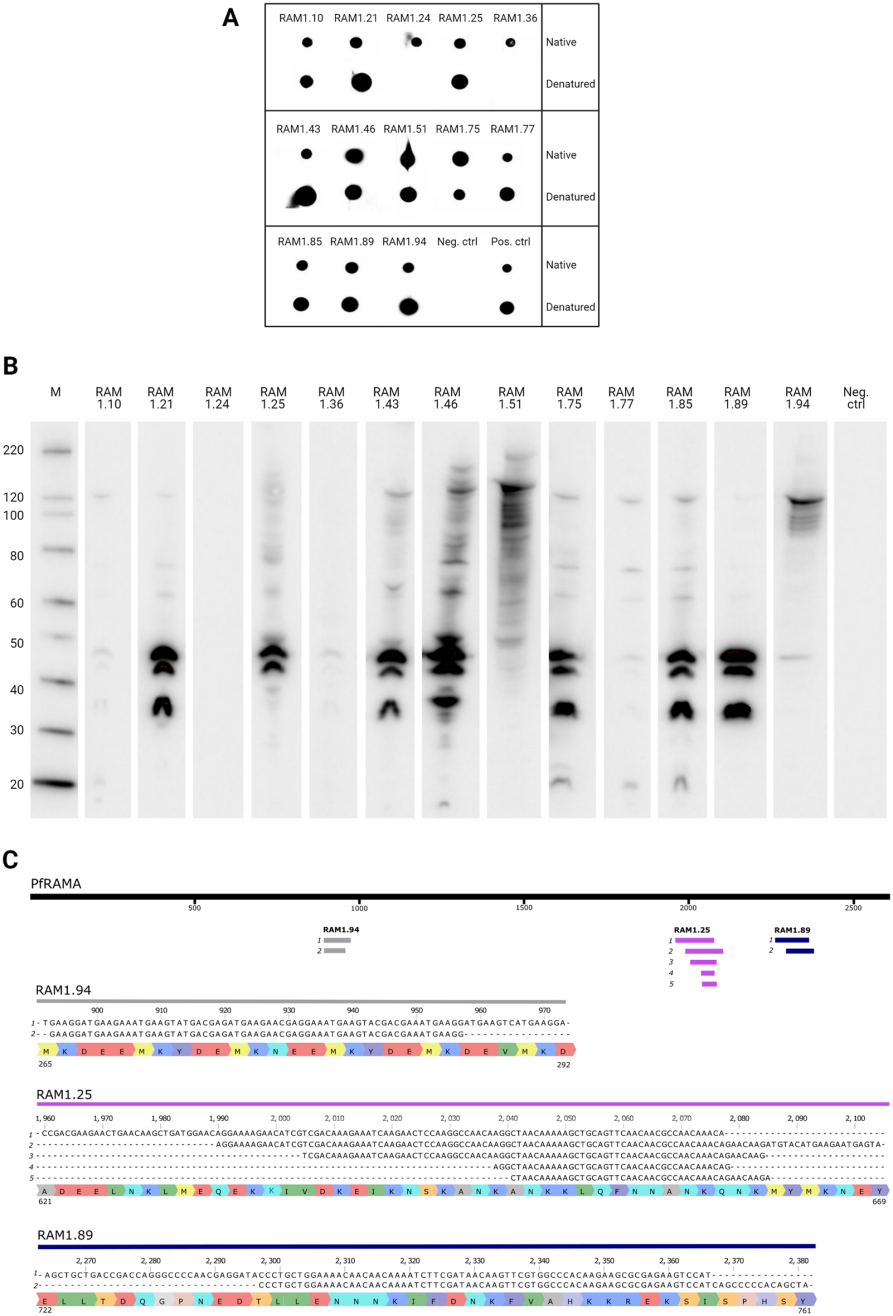
Assessing the binding domain of anti-PfRAMA mAbs. **(A)** Dot blot showing anti-PfRAMA mAbs binding to recombinant PfRAMA in native or denatured conditions. The negative control is the secondary anti-mouse IgG—HRP only, while the positive control is an anti-his(C-term)—HRP antibody. Unprocessed images of dot blots can be found in supplementary information Fig. S2**(B)** Western blot on lysates from a synchronized culture of schizont-infected erythrocytes, roughly 40 h post-infection. The western blot was probed with anti-PfRAMA mAbs, which were detected by a rabbit anti-mouse immunoglobulin—HRP conjugated antibody. Molecular weight marker (MagicMark) is shown on the left and the negative control (detection antibody only) on schizont lysates, is shown on the right. Unprocessed images of western blots can be found in supplementary information Fig. S3.**(C)** Gene fragments identified with mAbs RAM1.25, RAM1.89 and RAM1.94 aligned to PfRAMA. The PfRAMA gene is shown in black with numbered base pairs as an overview. The sequences of the gene fragments derived from panning with mAbs RAM1.94 (grey), RAM1.25 (purple), and RAM1.89 (blue) are aligned to the PfRAMA gene, displayed as colored lines. Below, a detailed sketch provides the exact sequences and the encoded amino acids. Two unique gene fragments were identified with RAM1.94, five with RAM1.25 and another two with RAM1.89. The gene fragments are numbered according to the clone number shown in Supplementary Fig. S4.

To obtain a better understanding of the anti-PfRAMA mAbs and their inability to efficiently inhibit *P. falciparum* growth, we examined their epitopes on PfRAMA. Dot blots were performed on native and denatured recombinant PfRAMA, to determine whether the mAbs recognized linear or conformational epitopes. The majority of the anti-PfRAMA mAbs did not discriminate between the two conditions, indicating that they target linear epitopes on PfRAMA. Only two mAbs, RAM1.24 and RAM1.36, seemed to target conformational epitopes, as binding only occurred in native conditions (Fig. 5A and Fig. S3). Furthermore, we observed that higher signal was often detected when the mAbs recognized the protein in denatured conditions as opposed to native conditions. This could indicate that the linear epitopes are more accessible in the denatured PfRAMA, thereby resulting in higher signal.

Previously, it has been shown that full-length PfRAMA undergoes proteolytic cleavage in the rhoptries to form a C-terminal product of 55–60 kDa^11^. The amount of C-terminal PfRAMA increases with time relative to the full-length protein, however, both are still present in late erythrocytic stages, whereas the N-terminal part is rapidly degraded during processing^11,16^. Antibodies to C-terminal PfRAMA have been associated with resistance to *P. falciparum* infection^16,19^. Thus, we wanted to investigate whether the anti-PfRAMA mAbs recognized only full-length PfRAMA or both forms, as it might be associated with their inability to inhibit *P. falciparum* growth efficiently.

By performing western blots under non-reducing conditions, anti-PfRAMA mAbs were probed onto parasite lysates from synchronized schizont-enriched cultures (Fig. 5B and Fig. S4). The majority of anti-PfRAMA mAbs recognized multiple bands at approximately 130-, 75-, 60-, 50-, 45- and 35 kDa, despite the molecular mass of full-length PfRAMA being 103.7 kDa^11^. RAM1.24 and RAM1.36, which seem to target conformational epitopes on PfRAMA, detected very few bands on the parasite lysates, which may be the result of the harsh sample treatment. We suggest that the 130 kDa band in our western blot is full-length PfRAMA running higher than its predicted size, and that the smaller molecular weight bands are products of proteolytic cleavage. Furthermore, we suggest that the C-terminal form of PfRAMA, which is highly abundant in the schizont stage, has an apparent size of 50 kDa, although previously reported to be 55–60 kDa^11,15,16^. Most mAbs recognized both full-length PfRAMA and the C-terminal form, indicating that they target an epitope present in the C-terminal part of PfRAMA. Of these mAbs, the growth inhibitory mAb RAM1.25 stands out, as the 35 kDa band was undetectable, indicating recognition of a unique epitope. RAM1.51 and RAM1.94 differ significantly from the other mAbs, as they mainly recognized full-length PfRAMA, suggesting that their epitope is present in the N-terminal part of PfRAMA.

Since the dot blot indicated that the epitope of RAM1.25 was linear, we continued our search for the epitope by generating a phage display gene fragment library. The library was produced by amplifying the gene encoding PfRAMA, followed by performing a DNaseI treatment to yield random DNA fragments. The fragments in the range of 50–250 bp were ligated into a phagemid vector enabling display of PfRAMA derived peptides by fusion to the phage coat protein pIII. After three rounds of panning on RAM1.25, we identified PfRAMA fragments that bound specifically to the anti-PfRAMA mAb and not to a control mAb (TC80.1) (Fig. S5). By sequencing the inserts, we identified 5 unique fragments of various sizes that all aligned to aa 621–669 in the PfRAMA sequence (Fig. 5C). The smallest PfRAMA fragment recognized by RAM1.25 was only 14 aa long and aligned to aa 647–660 (RAM1.25 sequence 4). This sequence represents the minimal overlap between all the identified sequences. We also panned the library on RAM1.89 and RAM1.94, as both mAbs seemed to recognize different epitopes on PfRAMA according to the western blotting analysis. RAM1.89 recognized aa 722–761 C-terminally of the epitope of RAM1.25 and RAM1.94 recognized aa 265–292 N-terminally of the epitope of RAM1.25 (Fig. 5C, Fig. S5). These epitopes correlate with the bands identified by the western blotting analysis on merozoite extracts (Fig. 5B). As control for unspecific binding to antibodies, three rounds of panning were also performed on the control mAb (TC80.1). Phage clones with high reactivity to the panning mAb and not to a control mAb were only found when panning with the PfRAMA specific mAbs (Figure S5). From this we can conclude that RAM1.25 recognizes a segment within the ‘pr fragment’ at the C-terminal end of PfRAMA, which previously has been suggested to harbor protective epitopes^16^. The two non-inhibitory mAbs on the other hand recognize epitopes either in the N-terminus part of PfRAMA (RAM1.94) or even more C-terminally (RAM1.89) than RAM1.25.

## Discussion

PfRAMA is a rhoptry bulb protein that is expressed before de novo formation of rhoptries. Full-length PfRAMA undergoes proteolytic cleavage in the rhoptries, after which the C-terminal part persists and the N-terminal part rapidly degrades^11^. As the protein is essential for parasite survival^13^ and polyclonal antibodies raised against full-length PfRAMA are able to inhibit parasite growth, the protein is considered a promising blood-stage vaccine candidate^17^.

Here, we examined a panel of anti-PfRAMA mAbs for their ability to inhibit *P. falciparum* growth in vitro. The vast majority of the mAbs were unable to neutralize the parasite, however, one anti-PfRAMA mAb was found to inhibit *P. falciparum* growth at high concentrations. In addition, enhanced neutralizing effects were observed when combining this mAb with anti-PfRh5 or anti-PfCyRPA mAbs. Our characterization further elucidated that the neutralizing anti-PfRAMA mAb targets a unique epitope in the C-terminus of PfRAMA.

PfRAMA comprises 861 amino acids with a predicted molecular weight of 103.7 kDa^11^. The protein is primarily hydrophilic, with three distinct highly charged repeat regions in the N-terminus^11^. A previous study have shown by western blotting on parasite lysates that full-length PfRAMA had an apparent size of 170 kDa, noticeably bigger than its predicted size^11^.

In this study, we analyzed full-length recombinant PfRAMA by western blotting and found no difference between the predicted molecular weight (124 kDa) and the apparent molecular weight of the protein. In our analysis of endogenous PfRAMA in parasite lysates, we did not detect a 103.7 kDa band as predicted. Instead, we suggest full-length endogenous PfRAMA to have an apparent size of 130 kDa. The discrepant results in size between our detection of PfRAMA and previous studies, which reported 170 kDa, is probably associated with different percentages of polyacrylamide gels and/or the type of running buffer^11,15^. Nevertheless, it is a consistent feature that endogenous PfRAMA runs higher than its predicted size (103.7 kDa), which may be the result of post-translational modifications such as phosphorylations. Furthermore, these post-translational modifications seem to occur only in the parasite, and not when expressing the protein recombinantly using the Expi293F expression system. As PfRAMA only contains one cysteine residue, it is unlikely that the size difference is associated with reduced or non-reduced conditions (results not shown).

The analysis of recombinant and endogenous PfRAMA identified pronounced processing of the protein. We detected an abundant amount of processed PfRAMA at approximately 50 kDa, in line with previous studies, which indicated accumulation of C-terminal PfRAMA of similar size in late erythrocytic stages^11,15,16^.

We successfully generated a panel of 13 anti-PfRAMA mAbs by hybridoma technology. These mAbs were able to detect endogenous PfRAMA by flow cytometry on fixed and permeabilized merozoites and by western blotting on schizont lysates. The majority of the mAbs recognized linear epitopes on PfRAMA, while only two mAbs, RAM1.24 and RAM1.36, seemed to recognize conformational epitopes. Both mAbs showed limited PfRAMA detection by flow cytometry- and western blotting analyses, which we expect is associated with disruption of the three-dimensional structure of the protein.

Despite recognizing the endogenous protein, only one anti-PfRAMA mAb, RAM1.25, was capable of inhibiting parasite growth. The same mAb synergized in combination with anti-PfRh5 or anti-PfCyRPA mAbs confirming previous studies showing synergistic interactions between polyclonal antibodies against these targets^17^. As high concentrations of RAM1.25 were required to observe only limited in vitro GIA, it is unlikely that the mAb would have any in vivo efficacy on its own. Previous studies in mice and Aotus monkeys have shown that passive transfer of mAbs targeting merozoite antigens were able to protect from infection. The outcome of the in vivo studies correlated with antibody potency in the GIA assay, creating an important link between in vitro and in vivo growth inhibition^24,30,31^. As our study showed that RAM1.25 synergized in combination with anti-PfRh5 or anti-PfCyRPA mAbs, we speculate that passive transfer of dual mAb combinations involving RAM1.25, might show efficacy in vivo. In our assessment of functionality, we also tried to mimic a polyclonal antibody response against PfRAMA by pooling all 13 anti-PfRAMA mAbs. No improvement in GIA was observed, which could suggest that PfRAMA contains many immunogenic epitopes that are not involved in any direct host interaction or that our panel of antibodies targets a similar area on the protein. As a former study had shown high levels of parasite neutralization using polyclonal antibodies against PfRAMA^17^, we were surprised that the vast majority of our mAbs were unable to exhibit a similar level of inhibition. It has previously been hypothesized that anti-PfRAMA antibodies can inhibit erythrocyte invasion by different mechanisms of action^16^. In our study, only direct inhibition was assessed by the GIA assay, but the parasite neutralization might be mediated through complement activation, antibody-dependent cellular inhibition or opsonic phagocytosis^32–35^. These possibilities remain to be examined.

Another feature that could influence the neutralizing capacity of anti-PfRAMA mAbs is the epitope specificity. Associations between antibody responses against C-terminal PfRAMA and malaria immunity have been observed in various cohorts^11,16,19,36^. One study identified the extreme C-terminus of PfRAMA as a protective epitope, as prevalence and magnitude of IgG1 and IgG3 antibodies specific to PfRAMA ‘fragment E’ (759–840 aa) were higher in clinical immune individuals compared to susceptible individuals living in an malaria endemic area in Vietnam^19^. A different study identified PfRAMA ‘fragment pr’ (582–767 aa) as a target of protective IgG1 responses in resistant individuals living in an endemic region in Western Kenya^16^. Despite these varying results, both studies concluded that antibodies specific to certain parts of PfRAMA were associated with resistance to *P. falciparum* infection^16,19^.

The identification and characterization of a growth inhibitory anti-PfRAMA mAb could elucidate the exact location of a protective epitope in the C-terminal part of PfRAMA. Here, we showed that RAM1.25 inhibits *P. falciparum* growth in vitro. A western blotting analysis on schizont lysates indicated that RAM1.25 recognized the abundant processed C-terminal form of PfRAMA of 50 kDa. We suggest the 45- and 35 kDa bands to be products of further processing of the 50 kDa PfRAMA, as the intensity of these bands was similar. The majority of the anti-PfRAMA mAbs recognized all three bands, but remarkably, RAM1.25 did not recognize the smallest fragment at 35 kDa. We further identified the minimal binding site of RAM1.25 by using a gene fragment library of PfRAMA. This experiment supported the western blotting analysis, showing that the epitope is located in the C-terminal ‘pr fragment’ but not in the extreme C-terminus^16^. This differs from the epitopes identified for the non-inhibitory mAbs.

In summary, our experiments demonstrated that anti-PfRAMA mAbs need to recognize a specific area of the antigen in order to exhibit any neutralizing effect on the parasite. We suggest that PfRAMA primarily contains immunogenic epitopes that elicit antibody responses unable of inhibiting parasite:host interactions. As we identified a PfRAMA segment that seems to harbor the epitope of the neutralizing and synergistic anti-PfRAMA mAb (RAM1.25), future work might entail translating this knowledge into the design of a novel blood-stage malaria vaccine. By using the identified peptides, it might be possible to generate an antibody response better at blocking erythrocyte invasion, compared to the response generated by the full-length protein. Our study also showed that the neutralizing effect of an anti-PfRAMA mAb was potentiated when combined with anti-PfRh5 or anti-PfCyRPA mAbs. Interestingly, a synergistic effect was only achieved when RAM1.25 was combined with a specific anti-PfCyRPA mAb, which we are yet to fully understand. Nevertheless, this study suggests a combined use of PfRAMA, PfRh5 and PfCyRPA antigens, or more likely certain fragments of these antigens, to improve the efficacy of blood-stage malaria vaccines.

## Methods

### Expression and purification of recombination PfRAMA

The plasmid encoding the *P. falciparum* rhoptry antigen PfRAMA-bio-his (Addgene, plasmid #50737) was a gift from Gavin Wright (Wellcome Sanger Institute, Cambridge, UK). The PfRAMA sequence was based on the 3D7 strain and comprised amino acids Y17-S840, where any predicted N-linked glycosylations were removed by substituting alanine for serine/threonine at these sites. Additionally, the vector included a 25 kDa C-terminal tag-region comprising ratCD4 (d3 + 4), a biotinylation sequence and a hexahistidine (His6) tag^21^.

The recombinant protein was expressed by Expi293F cells (Gibco), by transiently transfecting the PfRAMA-bio-his plasmid following the manufacturer’s instructions. The secreted protein was harvested three days post transfection and purified by immobilized metal affinity chromatography (IMAC) using a 5 mL HiTrap IMAC HP column charged with 0.1 M NiSO_4_ × 6 H_2_O on the ÄKTAxpress system (both GE Healthcare). PfRAMA was eluted in 500 mM imidazole in sodium phosphate buffer pH 7.4, immediately followed by buffer-exchanging into PBS using Amicon ultra centrifugal concentrators (Millipore). The quality of PfRAMA was evaluated by SDS-PAGE and western blotting using respectively InstantBlue staining (Expedeon) or an anti-his (C-term)-HRP conjugated antibody (Miltenyi Biotec) for detection.

### Mice immunizations

The mice immunizations were conducted in accordance with the Federation of European Laboratory Animal Science Associations (FELASA) guidelines and reported according to ARRIVE guidelines. Ethical approval was granted by the Danish Animal Experiment Inspectorate (approval number: 2018-15-0201-01541), an ethical committee under Danish veterinary and Food Administration. The study involved 6-week-old female BALB/c ByJR mice purchased from Janvier Labs. Five mice were immunized intramuscularly (IM) with 20 µg of PfRAMA formulated in 50% v/v AddaVax adjuvant (InvivoGen). Mice were anesthetized during the procedure using 3.5% isoflurane. This procedure was then repeated two more times at 2-week intervals. Two weeks after the last IM boost, the mice were given an intraperitoneal (IP) boost with PfRAMA in PBS, and three days later, the mice were euthanized using isoflurane and cervical dislocation. Spleens and blood samples were collected during this procedure.

### Production of monoclonal antibodies

After picking the best immune responders, hybridoma cell lines were generated by fusing splenocytes of immunized mice with myeloma cells (SP2/0-Ag14) following the instructions and materials from the ClonaCell-HY hybridoma cloning kit (Stemcell Technologies). Briefly, the fusion was facilitated by resuspending splenocytes and myeloma cells in polyethylene glycol (PEG). Hybridoma cell lines were harvested 14 days after the fusion and plated into 96-well culture plates in HT supplemented media. Screening of hybridoma cell lines producing antibodies specific to PfRAMA was performed by ELISA, as described previously^23,37^. By single cell sorting using the FACSMelody (BD), monoclonal PfRAMA-specific hybridoma cell lines were obtained.

### Antibody purification

Supernatants of hybridoma cell lines producing PfRAMA-specific mAbs were harvested until 150–200 mL was collected. The supernatants were filtered and subsequently manually purified using 1 mL self-packed protein G plus agarose columns (Pierce). The antibodies were eluted in 0.1 M glycine pH 2.8 and neutralized with Trizma hydrochloride solution 1 M pH 9. All antibodies were buffer-exchanged into PBS and RPMI 1640 using Amicon ultra centrifugal concentrators as per manufacturer’s instructions.

### Intracellular staining for PfRAMA in *P. falciparum* merozoites

Viable merozoites were isolated as described earlier^38^. The hemozoin crystals were removed by running the resuspended merozoites through magnetic LS columns (Miltenyi Biotec). The isolated merozoites were resuspended in cold 3% BSA in PBS and seeded into 96-well U-bottom plates at a density of approximately 4 × 10^5^ merozoites in 100 μL. Merozoites were fixed in 4% paraformaldehyde in PBS for 15 min, followed by a permeabilization step using 0.1% Triton X-100 in PBS for 10 min. After blocking the merozoites in 3% BSA in PBS overnight, they were stained with mouse anti-PfRAMA mAbs (25 µg/mL) at room temperature for 30 min. This was followed by incubating with 20 µg/mL of detection antibody (FITC conjugated horse anti-mouse IgG Antibody (H + L), Vector Laboratories). Nuclei were stained with 300 nM DAPI (4′6-diamidino-2-phenylindole) for 10 min. Merozoites were washed three times in 3% BSA in PBS and finally resuspended in 150 µL of the wash solution. Samples were analyzed using the CytoFLEX S flow cytometer (Beckman Coulter) and data analysis was conducted using FlowJo v10.7.1 software. The merozoites were gated by size and granularity, followed by gating on singlets and positive DAPI fluorescence. Lastly, histograms were generated and the geometric mean of the FITC intensity was calculated.

### In vitro growth inhibition activity (GIA) assay

The ability of antibodies to inhibit growth of *P. falciparum* 3D7 was assessed using the standardized one-cycle GIA assay, as previously described^39^. The assay was performed by mixing PfRAMA, PfCyRPA or PfRh5 specific antibodies with mid-trophozoite stages of *P. falciparum*. The assay was harvested after approximately 48 h culture, when the parasite were late-trophozoites to early schizonts. The parasite growth was determined by a biochemical assay, determining the amount of parasite lactate dehydrogenase, and the results were obtained by measuring the absorbance at 630 nm. The ability of an antibody to inhibit *P. falciparum* growth, was expressed as percent growth inhibition, calculated using the following equation:

$$GIA \% = 100 {-} \left[ {\frac{{\left( {A630\;Inhibited \;sample {-} A630\; RBCs \;only} \right) }}{{\left( {A630\; Uninhibited \;sample {-} A630\; RBCs \;only} \right)}} \times 100} \right]$$

The percent of GIA determined for the panel of antibodies was compared with positive and negative controls (normal growth medium, 5 mM EDTA, a growth inhibitory PfRh5-specific mAb (R5.016)^22^ and a non-inhibitory control mAb).

The theory of Bliss additivity was applied to assess synergy, when evaluating the effect of combining two antibodies. The mAb combinations were setup, either by mixing two antibodies in equimolar ratios, or by using a fixed concentration of one potent mAb and a dilution series of a less potent mAb. The predicted additive effect was calculated by Bliss additivity, as described previously^25,28^.

An isobologram analysis was also performed to assess the effect of combining antibodies. Dose–response curves were generated to obtain EC_30_ values of individual antibodies. The antibody interactions were assessed over a range of concentrations by a fixed-ratio method, using the following ratios between two antibodies (4:1, 3:2, 2:3 and 1:4)^29^. The interpolated EC_30_ values were used to calculate the 30% fractional inhibitory concentrations (FIC_30_), which then were used to plot an isobologram. A combination index was calculated by summing the FIC_30_ values derived from each of two antibodies in the given combination^17,29^.

To validate the results of the LDH measurements, light microscopy was employed to directly count the inhibition of parasite growth. In these assays the parasite growth inhibition capacity of the RAM1.25 mAb at 2000 µg/µL, and the synergistic effect of the combination of RAM1.25 mAb (2000 µg/µL) with CyP2.38 mAb (40 µg/µL) was analyzed. Two identical plates were set up in order to perform LDH assay and light microscopy counting in parallel. Each mAb or mAb combination was set up in triplicate in half-area flat bottom 96 well plates (Corning). The results are expressed as percent growth inhibition (previously described for the LDH assay). For the light microscopy, blood smears were prepared from each sample. Smears were air dried, fixed with methanol and stained with Giemsa (1:10 in RPMI) for 10 min. For each smear, the number of infected erythrocytes for every 300 erythrocytes was counted and used to calculate the percent growth inhibition relative to the growth of the parasite in normal growth medium (set as 100% growth).

### Dot blotting on native and denatured PfRAMA

The recombinant antigen PfRAMA-bio-his was diluted in PBS or PBS + 50 mM DTT at 10 µg/mL. For analysis under denatured conditions, the PfRAMA sample was heated to 95 °C for 5 min before spotting onto a 0.45 µm nitrocellulose membrane. The membrane was air-dried for 10 min followed by blocking in 5% skim milk in TBS + 0.05% Tween20 (TBS-T) for 1 h. After three washes with TBS-T, the membrane was immersed into separate anti-PfRAMA mAb solutions (5 µg/mL) in 2.5% skim milk in TBS-T for 1 h. The membrane was washed three times with TBS-T, followed by the addition of the detection antibody, anti-mouse IgG (γ-chain specific) HRP conjugated (Sigma), diluted 3000-fold in 2.5% skim milk TBS-T for 1 h. Finally, the membrane was washed three times with TBS-T, and the dot blot was developed with the KLP LumiGLO Reserve Chemiluminescent Substrates (SeraCare). The images were acquired using the ImageQuant Las4000 (GE Healthcare).

### Protein extraction from *P. falciparum* schizonts

Early *P. falciparum* 3D7 schizonts were isolated by magnetic-activated cell sorting (MACS) using a Vario-MACS magnetic separation unit and a MACS CS-column (both from Miltenyi Biotec)^40^. After the isolation, the infected erythrocytes were resuspended in complete medium and incubated at 37 °C for 3–4 h. The lysates were prepared essentially as described previously^41^. In brief, the isolated schizonts were washed once in PBS, followed by resuspending the pellet in 20 × volume of 0.06% (w/v) saponin in PBS and leaving the solution on ice for 20 min. The parasites were pelleted by centrifuging at 16,000×*g*, 4 °C for 5 min, followed by several wash steps in PBS. For protein extraction, the pellet was resuspended in 3 × ice-cold radioimmunoprecipitation assay (RIPA) buffer (Thermo Fisher Scientific) supplemented with a protease inhibitor cocktail (Roche Diagnostics). The solution was vigorously vortexed, before incubating the pellet on ice for 15 min. Finally, the lysates were cleared by centrifugation at 15,000×*g* for 15 min and the supernatant was stored at − 80 °C until use in SDS-PAGE.

### Western blotting analysis using schizont lysates

The protein concentration in schizont lysates was quantified using a BCA protein assay kit (Pierce) prior to loading on a gel. For SDS-PAGE, 30 µg of lysate was added in each lane of a NUPAGE 4–12% gradient Bis–Tris gel (Invitrogen) with MOPS running buffer. Samples were prepared in 6 × loading buffer under non-reducing conditions and heated to 70 °C for 10 min before loading. Using a wet-blotting system (Novex X-Cell II, Invitrogen), the proteins were transferred to a 0.45 µm nitrocellulose membrane. After blocking, PfRAMA was detected by our panel of mouse anti-PfRAMA mAbs (5 µg/mL) and incubated for 1 h. This step was followed by adding a secondary rabbit anti-mouse immunoglobulin—HRP conjugated antibody (DAKO) for 1 h. After washing the membrane, western blots were developed by LumiGLO peroxidase chemiluminescent substrate (SeraCare) and images were acquired using the ChemiDoc MP Imaging System (Bio-rad).

### Generation of PfRAMA gene fragment library and phage display selection with mAbs RAM1.25, RAM1.89 and RAM1.94

The gene encoding PfRAMA was amplified from the Addgene plasmid #50737 using Phusion HF PCR Mastermix (NEB) and purified using QIAquick PCR Purification Kit (QIAGEN). Next, 5 µg of PCR product was digested with 1 unit of DNaseI (NEB) for 1 min and 45 s. The yielded gene fragments were run on a 1% agarose gel and the smears located between 50 and 250 bp were cut out and purified using QIAX II Gel Extraction Kit (QIAGEN). The gene fragments were blunt end polished using Vent polymerase (NEB) and ligated into a linearized pHEN6 phagemid vector, cut at the restriction sites NotI and PstI (both Thermo Scientific). The phagemid vectors containing the PfRAMA gene fragments were transformed into the *E. coli* TG1 strain using electroporation (BioRad Gene Pulser, single pulse, 2.5 kV, 25 µF, 200 ohms).

M13KO7 helper phages (NEB) were added to the TG1 culture to produce functioning phages displaying PfRAMA peptide fragments on the pIII phage protein^42^. The phages were purified by precipitation using a 15% NaCl, 20% PEG10,000 solution (Sigma Aldrich). Phages were panned against the mAb of interest and a negative control mAb (TC80.1 mouse IgG1), which were bound to magnetic protein G beads (Dynabeads™ Protein G, Invitrogen). Phages were incubated with either RAM1.25, RAM1.89, RAM1.94 or TC80.1 coupled beads and washed to remove non-binding phages. The binding phages were eluted using a 0.2 M glycine solution pH 2.2 and allowed to reinfect new TG1 cells. The panning was repeated on the amplified phages two times, for a total of three pannings against the respective mAb. A negative selection strategy was performed in round two and three to remove nonspecific binders. This was done by panning the phages on protein G beads only, followed by panning on the mAb coupled beads.

After the last round of panning, the reinfected TG1 cells were screened by PCR and phage ELISA to verify insertion of a PfRAMA gene fragment and to confirm binding to the mAb of interest. The phage ELISA was performed by coating MaxiSorp flat-bottom 96-well plates with RAM1.25, RAM1.89, RAM1.94 or TC80.1 (10 μg/mL in PBS) overnight at 4 °C. Next, the plate was incubated for 1 h at RT with supernatant from a single colony culture expressing phages. Bound phages were detected with rabbit anti-fd phage polyclonal IgG (Sigma-Aldrich, B7786) followed by adding the secondary reagent, polyclonal goat anti-rabbit immunoglobulins—HRP conjugated (Agilent, P0448). The ELISA was developed using TMB PLUS2 substrate (Kementec) and the reaction stopped with 0.2 M H_2_SO_4_. The ELISA was read at 450 nm using the HiPo MPP-96 Microplate Photometer (Biosan). The signal from wells with no phages (coating mAb, anti-phage and anti-anti-phage HRP polyclonal) was defined as background and subtracted from the signals from the wells containing phages. Positive hits in the PCR and phage ELISA screenings were picked and sent for sequencing (Eurofins Genomics). The sequences were filtered for high-quality reads and sections localizing to the vector backbone of the pHEN6 plasmid were removed. Further, the parts of the sequences not aligning to PfRAMA were removed. The trimmed sequences were aligned to the PfRAMA encoding gene using the Benchling software.

### Ethics approval

The study involved mice work, which was conducted in accordance with the Federation of European Laboratory Animal Science Associations (FELASA) guidelines and granted ethical approval by the Danish Animal Experiment Inspectorate (approval number: 2018-15-0201-01541).

## Supplementary Information

Supplementary Figures.

## Data Availability

All data generated or analyzed during this study are included in this article and the supplementary information.
