# Supplementary material for: Enhancing neutralization of Plasmodium falciparum using a novel monoclonal antibody against the rhoptry-associated membrane antigen
Source: Sci Rep. 2022 Feb 23;12:3040. doi: 10.1038/s41598-022-06921-1 (PMC8866459; doi:10.1038/s41598-022-06921-1)
Supplement: Supplementary file 1 — Supplementary Figures. [file 41598_2022_6921_MOESM1_ESM.docx]

**Supplementary information**

**Manuscript:**

**Enhancing neutralization of *Plasmodium falciparum* using a novel monoclonal antibody against the rhoptry-associated membrane antigen**

Anne S. Knudsen^1^, Melanie R. Walker^1^, Judit P. Agullet^1^, Kasper H. Björnsson^1^, Maria R. Bassi^1^ and Lea Barfod^1*^

*^1^Centre for Medical Parasitology, Department of Immunology and Microbiology, Faculty of Health and Medical Sciences, University of Copenhagen, Copenhagen, Denmark*

**
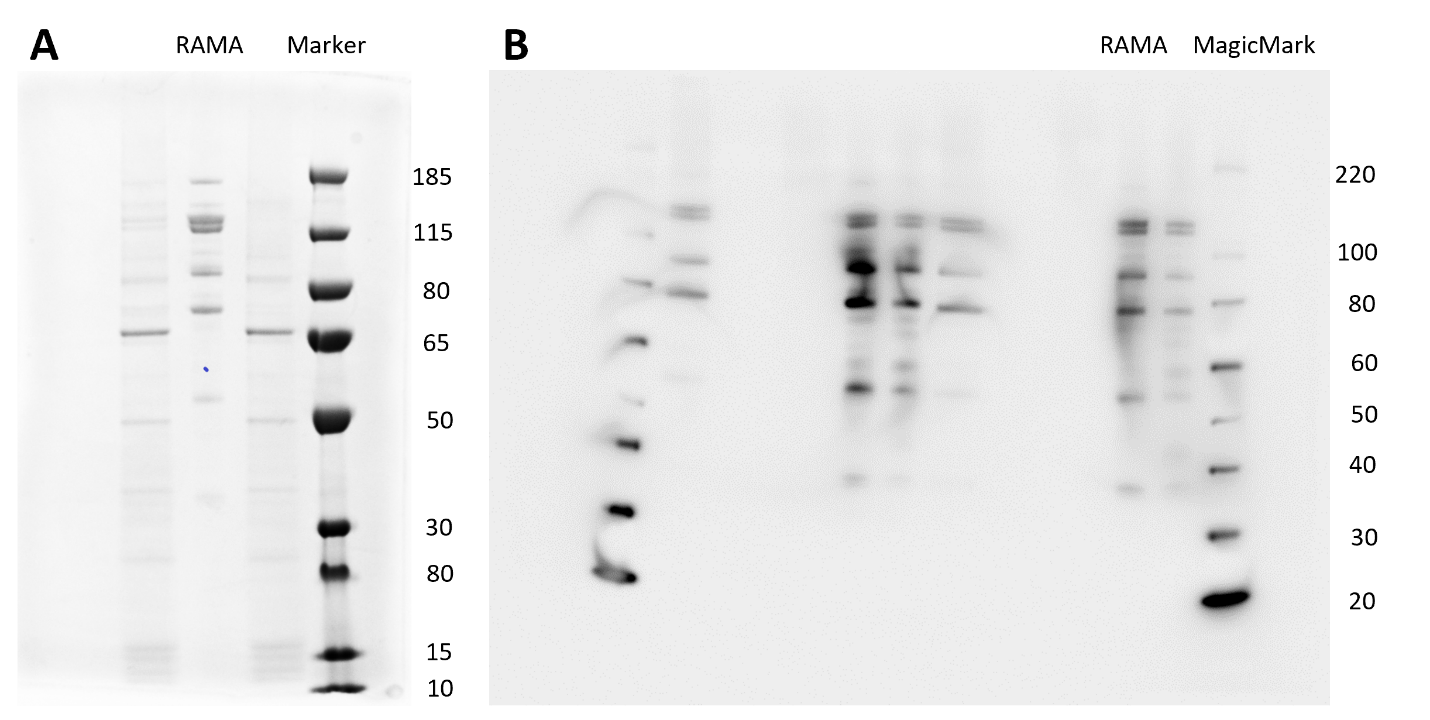
**

**Figure S1. Assessing the quality of PfRAMA (unprocessed images).**

**(A)** 1 µg of recombinant PfRAMA was resolved under non-reducing conditions by SDS-PAGE followed by Instant Blue^TM^ staining. The expected size is 124 kDa including the tag-region (25 kDa). **(B)** Western blotting analysis of 100 ng of recombinant PfRAMA under non-reducing conditions, resolved by SDS-PAGE, blotted and detected using anti-his(C-term)-HRP conjugated antibody.

**Figure S2: Correlation between LDH and light microscopy read-outs.** Average percent inhibition of *P. falciparum* growth measured either by the LDH assay or by counting Giemsa-stained slides by Light microscopy. Antibodies tested: RAM1.25 (2000µg/mL), CyP2.38 (40µg/mL), R5.016 (125µg/mL), RAM1.25 (2000µg/mL)+Ctrl. mAb (2000 µg/mL) and RAM1.25 (200µg/mL)+CyP2.38 (40µg/mL). Bars represent the standard error of the mean for light microscopy (vertical) or LDH assay (horizontal). The data presented is from one representative assay of three independent repeats.

**
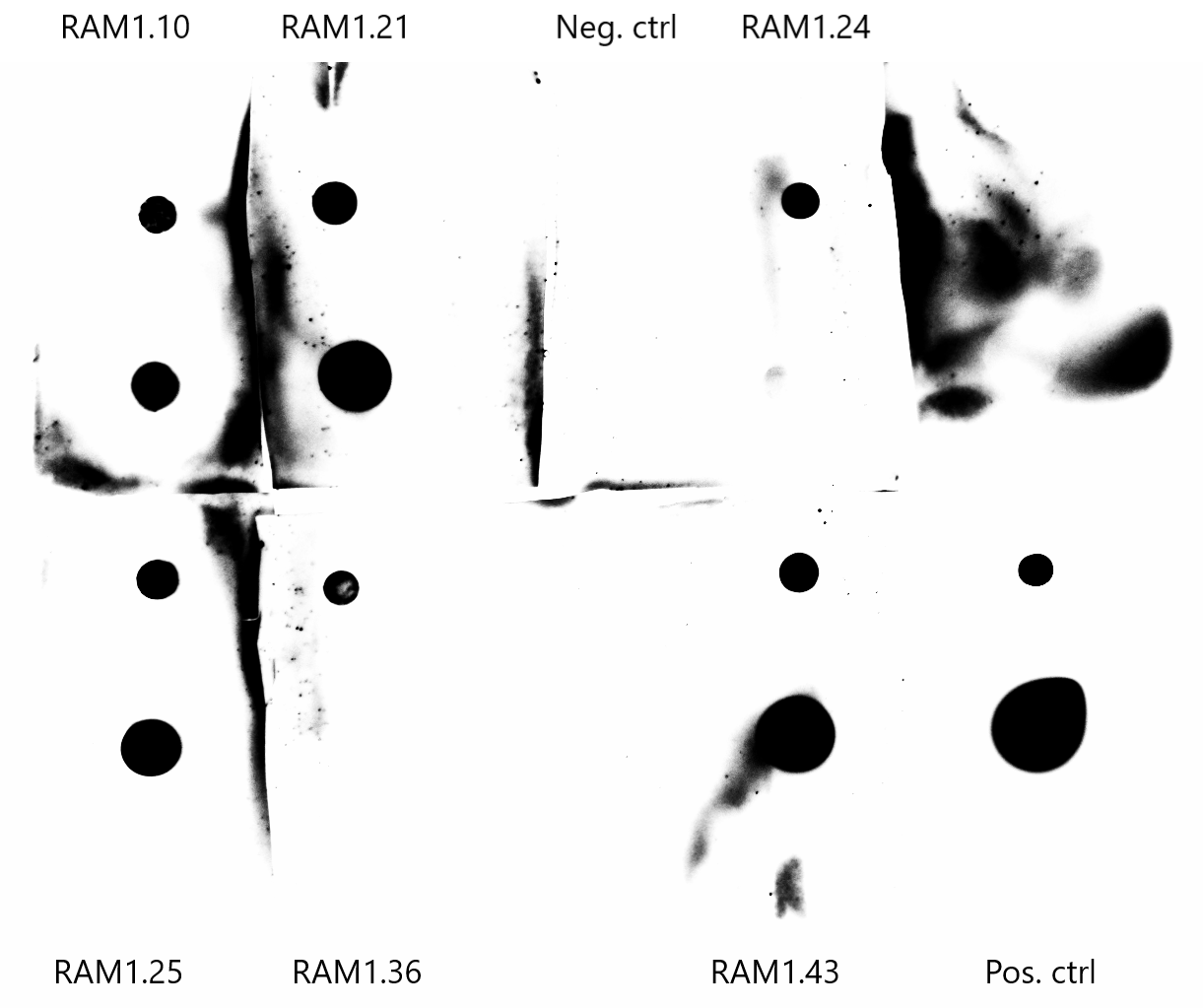
**

**
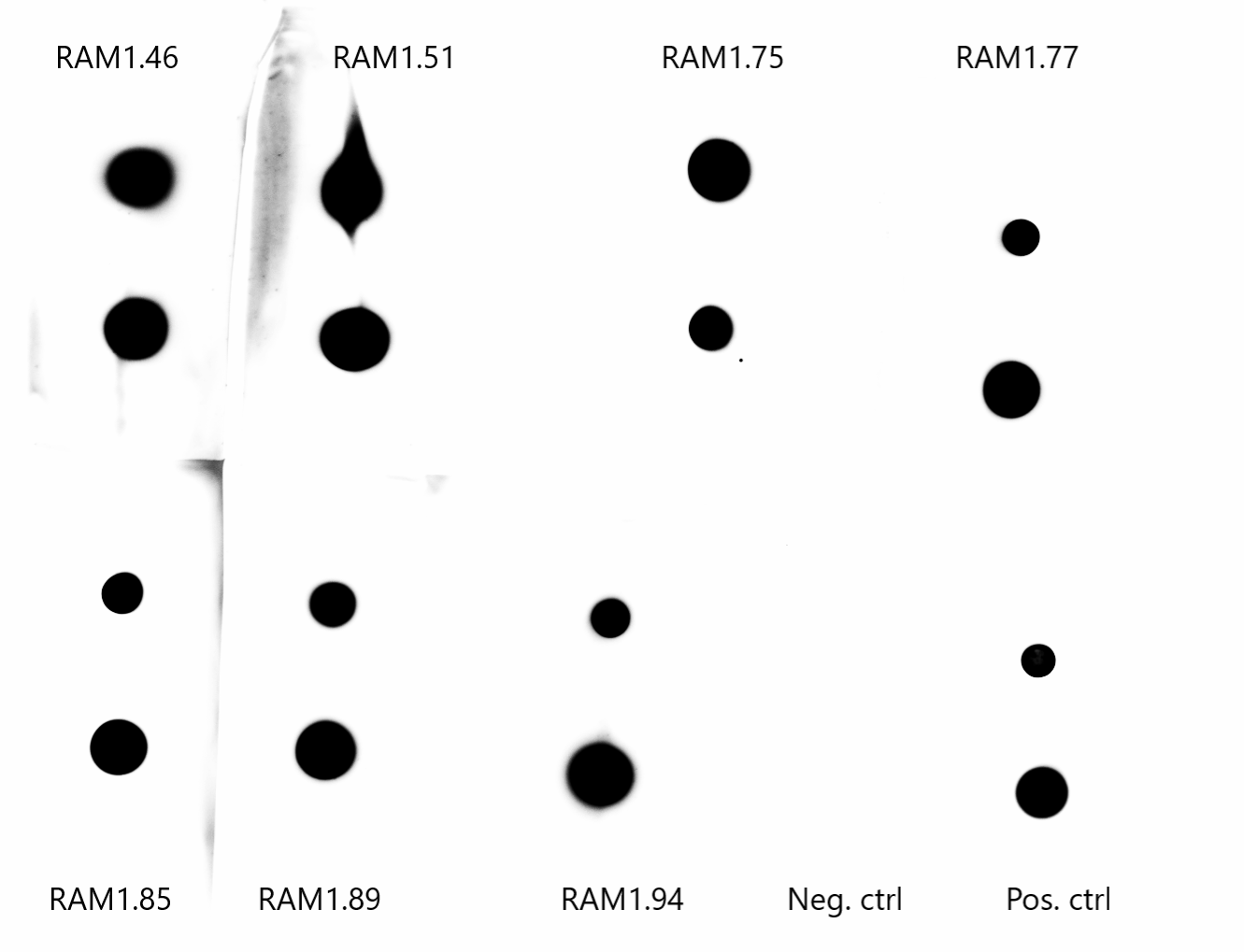
**

**Figure S3. Panel of anti-PfRAMA mAbs binding native or denatured PfRAMA (unprocessed images).**

Dot blot showing anti-PfRAMA mAbs binding to recombinant PfRAMA in native or denatured conditions, respectively the top or bottom dot. The negative control is the secondary anti-mouse IgG – HRP only, while the positive control is an anti-his(C-term) – HRP antibody.


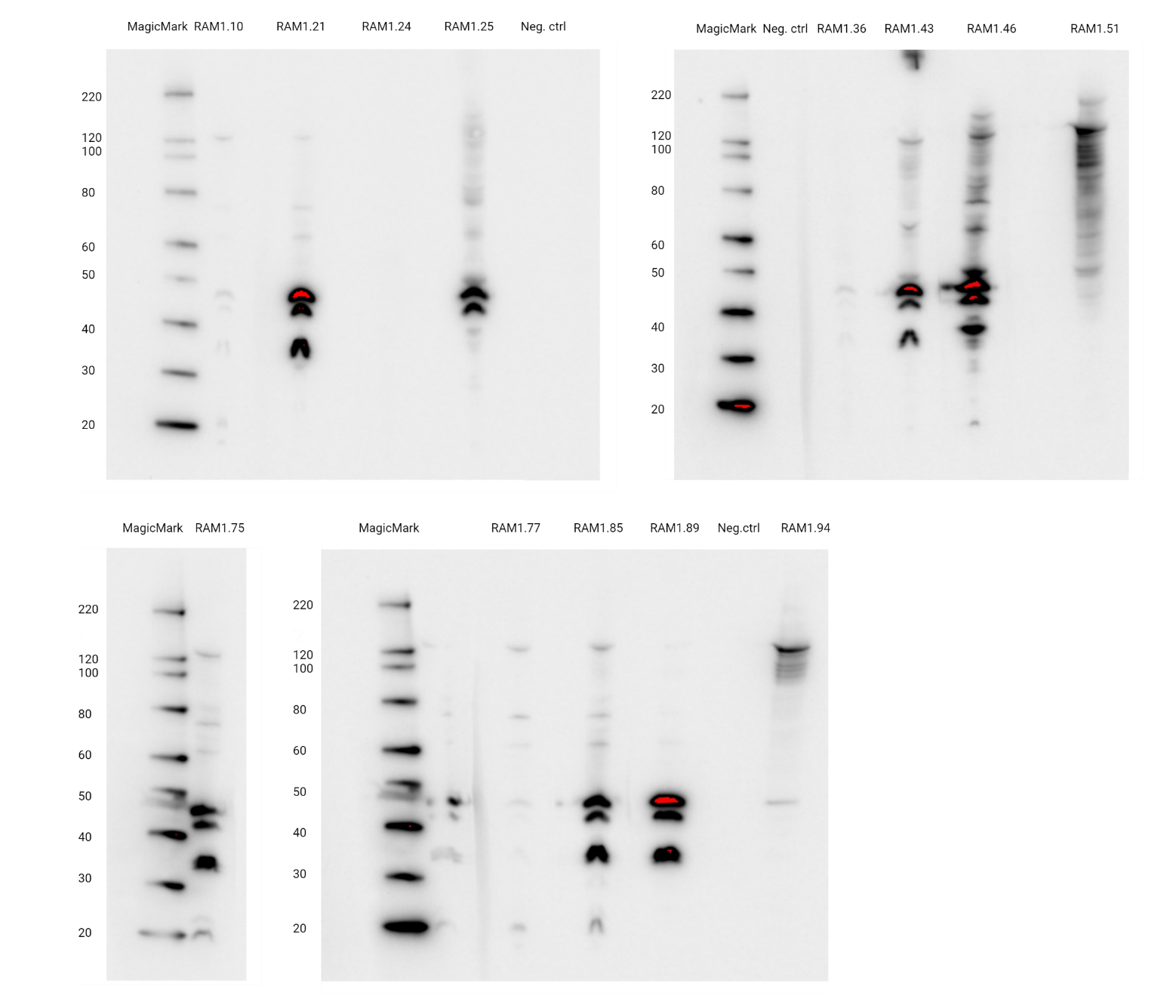


**Figure S4. Western blot on 3D7 schizont lysates probed with anti-PfRAMA mAbs (unprocessed images).**

Western blot on lysates from a synchronized culture of schizont-infected erythrocytes, roughly 40h post-infection. The western blot was probed with anti-PfRAMA mAbs, which were detected by a rabbit anti-mouse immunoglobulin – HRP conjugated antibody. Molecular weight marker (MagicMark^TM^) is shown on the left on each image. The negative control (detection antibody only) on schizont lysates is indicated on each image. The bottom images are from the same blot. Image of RAM1.75 needed to be repeated due to technical difficulties.


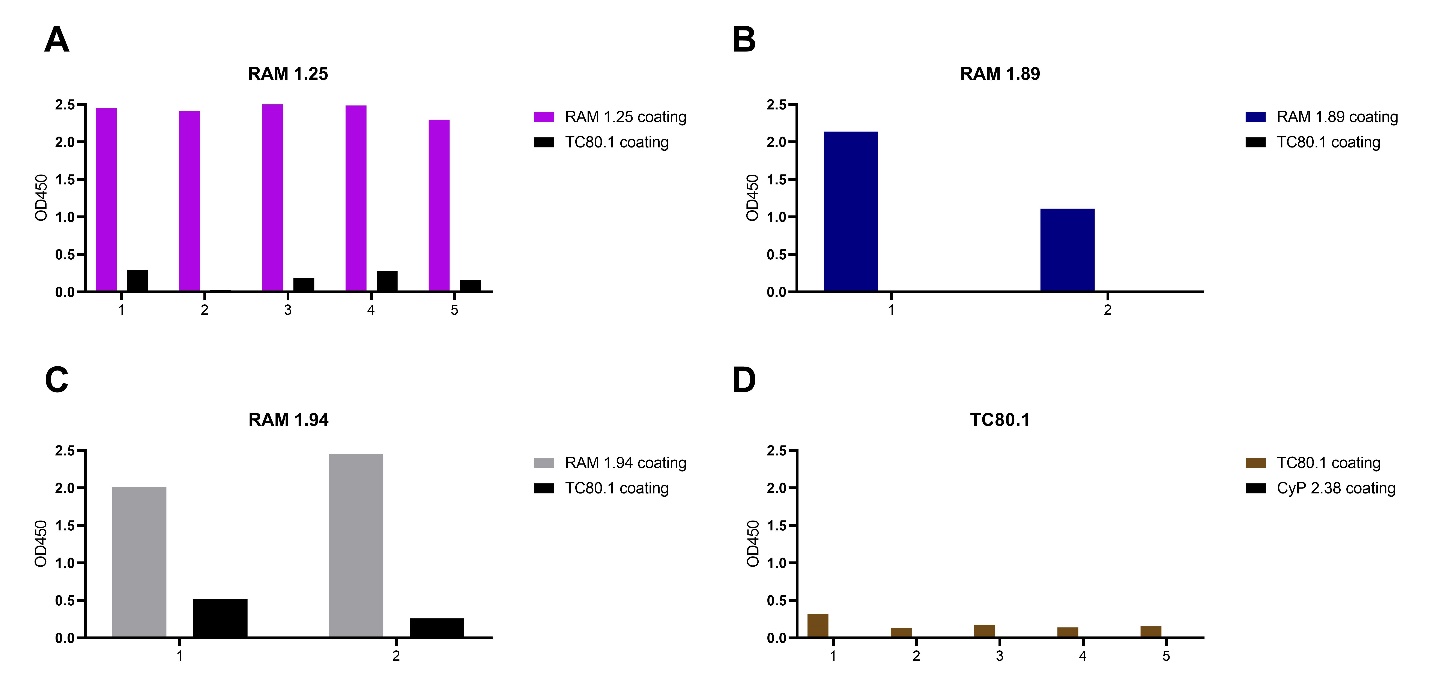


**Figure S5: Phage ELISA on unique clones obtained by panning a PfRAMA gene fragment library against anti-PfRAMA mAbs.** Single phage clones obtained from the different pannings were identified using phage ELISA. **(A)** Unique clones identified from RAM1.25 panning, **(B)** unique clones identified from RAM1.89 panning, **(C)** unique clones identified from RAM1.94 panning and **(D)** unique clones identified from TC80.1 panning. The phage ELISA was coated with 10 µg/mL of the mAb of interest. This was followed by incubating with individual phages obtained after the third round of panning and sequencing the phagemid vector to determine the PfRAMA fragment sequence. A positive signal was acquired by adding rabbit anti-fd phage polyclonal IgG (Sigma-Aldrich, B7786) followed by goat anti-rabbit immunoglobulins HRP conjugated polyclonal antibodies (Agilent, P0448). The y-axis shows the OD450 with background subtracted. The x-axis shows the unique phage clone number. TC80.1 was used as a negative control for phages panned against anti-PfRAMA mAbs. mAb CyP2.38 was used as a negative control for phages panned against the TC80.1 mAb. The phage clone numbers correspond to the gene fragment numbers shown in Figure 5C. Values ≤ 0 cannot be seen on the graph, which is the case for some of the negative controls.
